# Differentiating between visual hallucination-free dementia with Lewy bodies and corticobasal syndrome on the basis of neuropsychology and perfusion single-photon emission computed tomography

**DOI:** 10.1186/s13195-014-0071-4

**Published:** 2014-12-05

**Authors:** Michael R Misch, Sara Mitchell, Philip L Francis, Kayla Sherborn, Katayoun Meradje, Alicia A McNeely, Kie Honjo, Jiali Zhao, Christopher JM Scott, Curtis B Caldwell, Lisa Ehrlich, Prathiba Shammi, Bradley J MacIntosh, Juan M Bilbao, Anthony E Lang, Sandra E Black, Mario Masellis

**Affiliations:** L.C. Campbell Cognitive Neurology Clinic, Sunnybrook Health Sciences Centre, Room A4 42, 2075 Bayview Avenue, Toronto, ON M4N 3M5 Canada; Department of Medical Biophysics, Sunnybrook Health Sciences Centre, University of Toronto, Room A4 42, 2075 Bayview Avenue, Toronto, ON M4N 3M5 Canada; Department of Nuclear Medicine, Sunnybrook Health Sciences Centre, University of Toronto, Room A4 42, 2075 Bayview Avenue, Toronto, ON M4N 3M5 Canada; Neuropsychology Clinic, Sunnybrook Health Sciences Centre, Room A4 42, 2075 Bayview Avenue, Toronto, ON M4N 3M5 Canada; Department of Pathology, Sunnybrook Health Sciences Centre, University of Toronto, Room A4 42, 2075 Bayview Avenue, Toronto, ON M4N 3M5 Canada; Morton and Gloria Shulman Movement Disorders Clinic and the Edmond J. Safra Program in Parkinson’s Disease, Toronto Western Hospital, University Health Network, Toronto, Canada; Department of Medicine (Neurology), Brain Sciences Research Program, Sunnybrook Health Sciences, Centre University of Toronto, Room A4 42, 2075 Bayview Avenue, Toronto, ON M4N 3M5 Canada; Cognition & Movement Disorders Clinic, Sunnybrook Health Sciences Centre, Room A4 42, 2075 Bayview Avenue, Toronto, ON M4N 3M5 Canada; Neurogenetics Section, Centre for Addiction and Mental Health, University of Toronto, Room A4 42, 2075 Bayview Avenue, Toronto, ON M4N 3M5 Canada

## Abstract

**Introduction:**

Dementia with Lewy bodies (DLB) and Corticobasal Syndrome (CBS) are atypical parkinsonian disorders with fronto-subcortical and posterior cognitive dysfunction as common features. While visual hallucinations are a good predictor of Lewy body pathology and are rare in CBS, they are not exhibited in all cases of DLB. Given the clinical overlap between these disorders, neuropsychological and imaging markers may aid in distinguishing these entities.

**Methods:**

Prospectively recruited case–control cohorts of CBS (*n* =31) and visual hallucination-free DLB (*n* =30), completed neuropsychological and neuropsychiatric measures as well as brain perfusion single-photon emission computed tomography and structural magnetic resonance imaging (MRI). Perfusion data were available for forty-two controls. Behavioural, perfusion, and cortical volume and thickness measures were compared between the groups to identify features that serve to differentiate them.

**Results:**

The Lewy body with no hallucinations group performed more poorly on measures of episodic memory compared to the corticobasal group, including the delayed and cued recall portions of the California Verbal Learning Test (F (1, 42) =23.1, *P* <0.001 and F (1, 42) =14.0, *P* =0.001 respectively) and the delayed visual reproduction of the Wechsler Memory Scale-Revised (F (1, 36) =9.7, *P* =0.004). The Lewy body group also demonstrated reduced perfusion in the left occipital pole compared to the corticobasal group (F (1,57) =7.4, *P* =0.009). At autopsy, the Lewy body cases all demonstrated mixed dementia with Lewy bodies, Alzheimer’s disease and small vessel arteriosclerosis, while the corticobasal cases demonstrated classical corticobasal degeneration in five, dementia with agyrophilic grains + corticobasal degeneration + cerebral amyloid angiopathy in one, Progressive Supranuclear Palsy in two, and Frontotemporal Lobar Degeneration-Ubiquitin/TAR DNA-binding protein 43 proteinopathy in one. MRI measures were not significantly different between the patient groups.

**Conclusions:**

Reduced perfusion in the left occipital region and worse episodic memory performance may help to distinguish between DLB cases who have never manifested with visual hallucinations and CBS at earlier stages of the disease. Development of reliable neuropsychological and imaging markers that improve diagnostic accuracy will become increasingly important as disease modifying therapies become available.

## Introduction

Dementia with Lewy Bodies (DLB) and Corticobasal Syndome (CBS) are atypical parkinsonian disorders associated with fronto-subcortical and posterior cognitive dysfunction. Pathologically, DLB is an α-synucleinopathy with many cases having concomitant Alzheimer’s pathology [1]. CBS is more heterogeneous with the following underlying pathological substrates observed: corticobasal degeneration (CBD) , progressive supranuclear palsy (PSP), frontotemporal lobar degeneration (FTLD)-Tau (Pick’s disease) and FTLD-Ubiquitin/TAR DNA binding protein (TDP-43), Alzheimer’s disease (AD) and, rarely, DLB [2–6]. In a small case series of pathologically proven CBD, one patient had a differential diagnosis of CBS versus DLB and was eventually confirmed to have CBD at autopsy [7]. Clinically, DLB is characterized by recurrent visual hallucinations, spontaneous parkinsonism, fluctuating attention and alertness as well as executive and visuospatial dysfunction [8,9]. ‘Classical’ CBS is characterized by asymmetric rigidity, apraxia, dystonia, myoclonus, alien-limb phenomenon and/or cortical sensory loss [10]; however, dementia is often the initial presentation and classical features may emerge only later in the disease course [11]. While the two disorders can usually be distinguished on clinical grounds in the mid-stages of the disease course, the early stages often have overlapping clinical features and accurate diagnosis is more challenging especially when not all features are present.

The diagnostic criteria for DLB have demonstrated greater specificity (95%) than sensitivity (83%) [12]; other studies have reported even less successful figures [13,14]. While visual hallucinations are exceptionally rare in CBS [15], they have been shown to be the most sensitive and specific predictor of DLB pathology [16]. However, the prevalence of visual hallucinations ranges between 30% to 80% of pathologically proven DLB cases, indicating a low negative predictive value [9]. Recognizing and diagnosing DLB presenting without visual hallucinations early on and distinguishing it from CBS and other atypical parkinsonian syndromes has direct clinical relevance. For example, the symptoms of DLB have been shown to respond well to cholinesterase inhibitors [17,18], while patients with frontotemporal dementia spectrum disorders that includes CBS, do not benefit cognitively and may be more prone to behavioural disturbance on this class of drugs [19]. As such, additional data made available by neuroimaging and neuropsychological assessment may help to best differentiate DLB without visual hallucinations from CBS and this could allow clinicians to better target symptomatic therapy and avoid unwanted side effects. Furthermore, understanding differences between these two parkinsonian disorders using neuroimaging and neuropsychological evaluation may also shed light onto pathological correlates.

The objectives of this study were to compare: 1) the initial clinical and standardized neuropsychological and neuropsychiatric profile of a prospective cohort of 30 DLB patients with no history of visual hallucinations (visual hallucination-free [VHF]-DLB) and 31 CBS patients ascertained from both a movement disorders clinic and a cognitive neurology clinic; and 2) the perfusion single-photon emission computed tomography (SPECT) and structural magnetic resonance imaging (MRI) imaging features of CBS and VHF-DLB patients to elucidate functional and structural imaging findings that most reliably distinguish the two groups.

## Methods

### Participants

Thirty participants meeting clinical criteria for possible (n =10) or probable DLB (n =20) [9] without any history of visual hallucinations were recruited through the Linda C. Campbell Cognitive Neurology Research Unit at Sunnybrook Health Sciences Centre. Thirty-one participants with a clinical diagnosis of CBS according to diagnostic criteria proposed by Boeve *et al*. [20] were recruited through the former clinic and the Movement Disorders Centre at the Toronto Western Hospital, University Health Network. Diagnoses were made by consensus agreement by at least two reviewing neurologists with expertise in neurodegenerative diseases (MM, AEL and/or SEB). This study also included 42 healthy controls with available SPECT data; 30 of them were matched to the VHF-DLB group (11 were unique to this group and 19 overlapped with the CBS control group) while 31 were matched to the CBS group (12 were unique to this group and 19 overlapped with the VHF-DLB control group). Controls were selected to match as closely as possible for age, sex and years of education for each patient group. Subjects had to have a SPECT scan completed and available for analysis to be included in the study. Subjects needed to be within the age range of 40 to 90, have contact with a primary caregiver on at least four days per week, were sufficiently literate and fluent in English, and their SPECT and neuropsychological evaluations were completed within a consecutive three-month period. Exclusion criteria were: presence of secondary/reversible causes of dementia which were untreated, concomitant neurological or psychiatric illness/substance use and abuse, history of significant head trauma, as well as lesions on MRI indicating another pathological condition. The majority of the VHF-DLB, CBS and normal controls were recruited and monitored as part of the Sunnybrook Dementia Study (ClinicalTrials.gov identifier: NCT01800214), a prospective longitudinal study of dementia and ageing. Seven of the VHF-DLB patients were recruited through a prospective pharmacogenetic study of cholinesterase inhibitor response in Lewy body spectrum disorders (ClinicalTrials.gov identifier: NCT01944436), which employed similar imaging and clinical assessments as the Sunnybrook Dementia Study. Both studies were approved by the local Research Ethics Boards at Sunnybrook Health Sciences Centre and the Toronto Western Hospital, University Health Network. Written informed consent was obtained from the participants or their substitute decision makers in accordance with the Declaration of Helsinki.

### Neuropsychological, neuropsychiatric and functional measures

Neuropsychological tests assessing general intelligence and cognition included Folstein’s Mini-Mental State Examination (MMSE) [21]; the Mattis Dementia Rating Scale (DRS) [22]; the Clock Drawing Test [23]; the National Adult Reading Test-Revised (NART-R) [24]; and Raven’s Progressive Matrices [25]. Tests assessing learning and episodic verbal memory included the California Verbal Learning Test (CVLT) [26], while the visual reproduction subtest of the Wechsler Memory Scale-Revised (WMS-R) assessed visual memory [27]. Measures of language function and naming included: the Boston Naming Test (BNT) [28]; semantic/categorical fluency [29]; and the comprehension subscale of the Western Aphasia Battery (WAB) [30]. Initially, the full WAB was given to all patients, but in the last few years it has only been administered if there was anomia detected on the BNT [30]. Facial and limb praxis was assessed using the WAB praxis subscale [30]. Attention and working memory was assessed using the Forward and Backward Digit Span tests from the WMS-R [27,31]. Several assessments of executive function were employed including: phonemic (F-, A-, and S-word) fluency [27,29]; the Trail Making Test A and B (TMT-A and -B) [27]; and the Wisconsin Card Sort Test (WCST) [32]. Visuospatial function was assessed using the Rey-Osterrieth Complex Figure Test [27,33,34] and the Benton Line Orientation task, which is motor-free and assesses visuospatial orientation and attention [27]. Behavioural function was investigated using the Neuropsychiatric Inventory (NPI-12) [35]. Severity of depressive symptoms was assessed using the Cornell Scale for Depression in Dementia (CSDD) [36]. Functional assessment was performed using the Disability Assessment for Dementia (DAD), which assesses both basic and instrumental activities of daily living including subcomponents of initiation, planning and performance [37].

### Brain SPECT acquisition and processing

SPECT imaging employed a triple-head gamma camera (Prism 3000XP; Phillips Medical Systems Inc., Cleveland, OH, USA) and was performed between 30 and 120 minutes after injection of 20 mCi (740 MBq) of Technetium-99 m ethyl cysteinate dimer (99mTc-ECD SPECT). Patients were asked to rest with their eyes open during the injection phase in a quiet room. A total of 120 views were acquired uniformly over 360 degrees using all three detectors fitted with ultra-high resolution fan-beam collimators. Each view consisted of a 128 × 128 pixel image. Imaging time was 19 minutes. A ramp-filtered back-projection algorithm followed by a three-dimensional restoration post-filter was employed for image reconstruction (Wiener filter, multiplier 1.0). Reconstructed image resolution was 9.7 mm full width at half maximum (FWHM). Ellipses were fit to the approximate location of the head outline in each transaxial image and a calculated attenuation correction applied [38]. Voxel dimensions were 2.18 × 2.18 × 3.56 mm.

### Brain MRI acquisition, processing and region of interest cortical volumetric/thickness assessment

#### MRI acquisition

Structural MRI was obtained in 29 of the 31 CBS patients and 25 of the 30 VHF-DLB patients using a standard protocol. Images were acquired on a 1.5 T Signa MR imager (GE Medical Systems, Milwaukee, WI, USA) and consisted of the following acquisitions: 1) T1-weighted (axial three-dimensional spoiled gradient (SPGR) echo, with echo time (TE) 5 ms, repetition time (TR) 35 ms, flip angle 35°, number of excitations (NEX) 1, field of view (FOV) 22 × 16.5 cm, in-plane resolution 0.859 × 0.859 mm and slice thickness 1.2 to 1.4 mm); 2) proton-density (PD); and 3) T2-weighted images (interleaved axial spin echo, with TEs 30 and 80 ms, TR 3 s, NEX 0.5, FOV 20 × 20 cm, in-plane resolution 0.781 × 0.781 mm and slice thickness 3 mm).

#### Brain extraction and automated tissue segmentation

Twenty-one of the 29 CBS scans and 23 of the 25 VHF-DLB scans were of sufficient quality to undergo semi-automated image analysis. Poor image quality was primarily due to head motion artifacts. Brain extraction and automated tissue segmentation were based on previously described methods [39,40]. Images were co-registered to the T1-weighted image using the Functional Magnetic Resonance Imaging of the Brain (FMRIB) Software Library’s (FSL) flirt tool and a normalised mutual information cost function [41]. Proton density (PD)/T2 images were used collectively to extract brain and subdural/ventricular cerebrospinal fluid (CSF), then the masked T1 was segmented using a T1-based protocol whereby local intensity histograms are fitted to four Gaussian curves to derive cut-offs for classifying each voxel as white matter, grey matter or CSF [39]. This is important for calculating the total intracranial volume in correcting for head size, especially in focal atrophy syndromes, such as CBS. Lesion Explorer was then used to further segment tissue that appears hyperintense on PD and T2 and a trained rater manually checked the lesion mask and corrected false positives/negatives [40]. This lesion mask was then overlaid onto the segmented brain to identify the four lesion classes (periventricular subcortical hyperintensities and black holes, as well as deep subcortical hyperintensities and black holes) based on three-dimensional location and T1 intensity [40].

#### FreeSurfer cortical thickness and volume calculations

The FreeSurfer cortical parcellation procedure was subsequently applied to the fully segmented Lesion Explorer scans. The boundary between white and grey matter was corrected for topological defects [42] and deformed outward in order to locate the pial surface and achieve the final thickness estimations [43]. A region of interest (ROI) gyral atlas was then applied to obtain cortical thickness (mm) and volume (mm^3^) measurements in 34 bilateral gyral ROIs [44].

### Statistical parametric mapping SPECT analysis

SPECT scans were converted to Analyze 7.5 format. Statistical Parametric Mapping version 5 (SPM5, Wellcome Department of Imaging Neuroscience, University College London, London, UK) was used for all imaging processing. Images were spatially normalised to a standard SPECT template in Montreal Neurological Institute (MNI) space [45] with re-sampling of voxel dimensions of 2 × 2 × 2 mm. Images were then smoothed spatially using an isotropic Gaussian kernel (12 mm FWHM). Proportional scaling was used to normalise image intensity values between subjects, thus reducing the chances that inter-subject variability in cerebral tracer uptake would influence regional perfusion changes. The cerebellum is frequently used to normalise SPECT counts in studies of dementia [45] and was shown to be the region of choice for normalisation in DLB and AD [46]. However, crossed cerebellar diaschisis may lead to relative differences in perfusion between the left and right cerebellar hemispheres, and if the whole cerebellum is used as the reference region in these cases, regional cerebral blood flow (rCBF) may be miscalculated. We, therefore, applied the following rule: if there was more than a 5% difference in counts between left and right cerebellar hemispheres, the hemisphere with the higher perfusion count was used as the reference region. If the hemispheric difference was less than 5% then the average count in the whole cerebellum was used as the reference region.

Voxel-by-voxel analyses were performed using unpaired t-tests to compare CBS to controls, and VHF-DLB to controls. Covariates were incorporated if they were found to be significantly different between groups. We reported significance using a voxel-wise *P*-value threshold (*P* <0.05) corrected for multiple comparisons and an extent threshold of at least 20 contiguous voxels (k_E_ ≥20). Our correction methodologies included controlling the family-wise error (FWE) rate [47] and controlling the false discovery rate (FDR) [48]. Controlling the FWE rate is more conservative, but is known to be associated with type II errors. A whole brain mask was used to exclude extracranial voxels from the analysis. The maximal peak coordinates of the perfusion differences were converted to Talairach space using the Yale Non-linear MNI to Talairach Converter [49,50]. These converted coordinates were translated into anatomical brain regions and Brodmann Areas (BAs) using Talairach Daemon Client [51,52].

### Region of interest SPECT method

**Figure 1:**
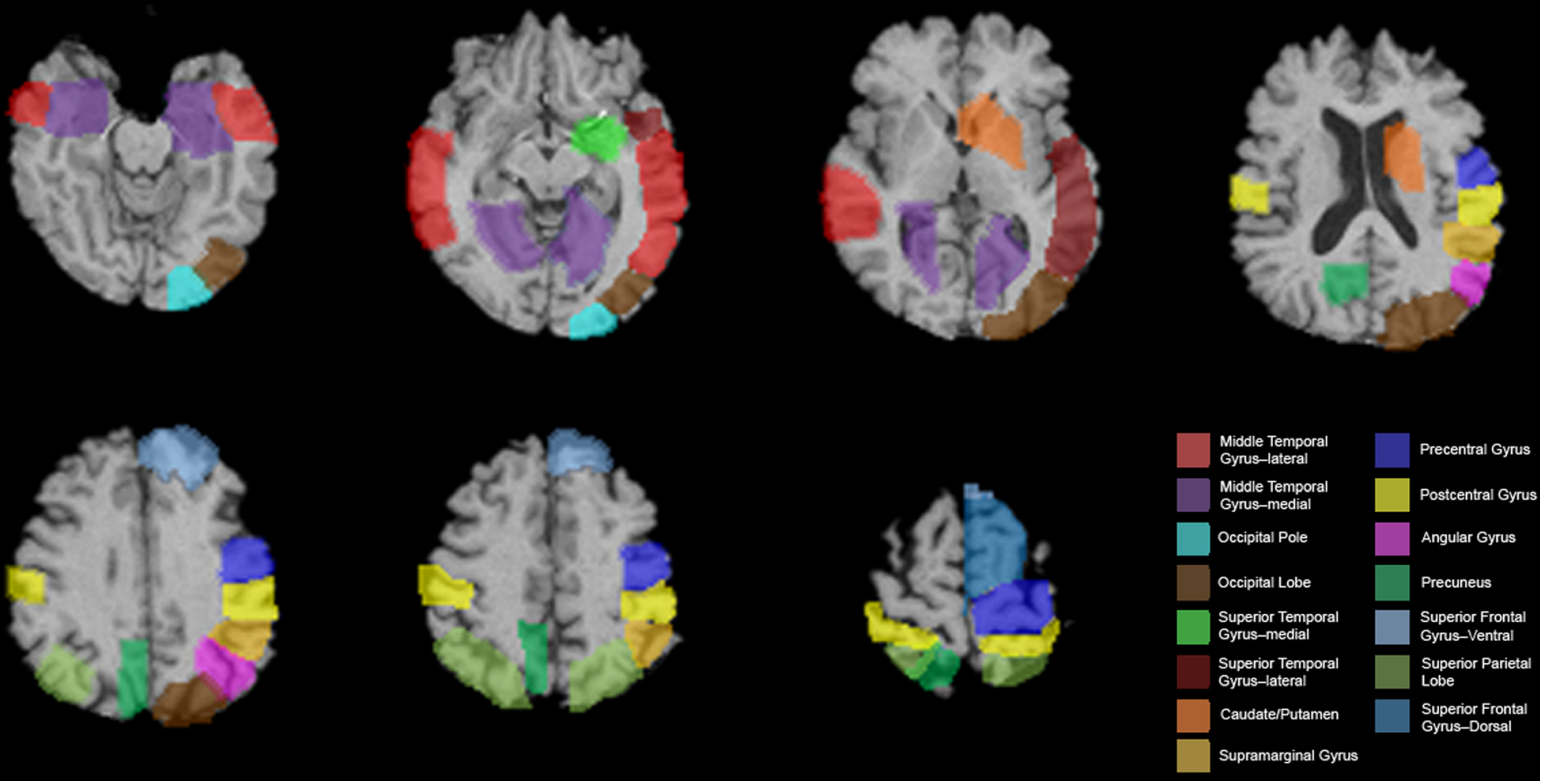
**Regions of interest (ROIs) that show differential perfusion in the case versus control analyses.** Views are shown in standard radiological orientation.

The second phase of the SPECT imaging analyses set out to identify regions of perfusion difference between the VHF-DLB group and the CBS group. A ROI analysis was performed using selected candidate brain regions that were found to show reduced perfusion in the case versus control analysis, but that did not overlap between the CBS and VHF-DLB groups (Figure 1). Reconstructed SPECT images were co-registered to a template that was an average of 14 healthy, elderly control scans. A T1-weighted MRI with dimensions similar to the SPECT template was the source of 79 bilateral ROIs as previously described [53]. To obtain ROI intensity values, we used a common transformation to move from the SPECT template space to MRI space. The cerebellum was used as the reference region in a manner similar to that described in the SPM analysis to generate semi-quantitative perfusion ratios in each ROI [53].

### Demographic, clinical, neuropsychological, SPECT ROI and MRI ROI cortical volumetric and thickness data analysis

Statistical analysis of demographic, clinical, neuropsychological and ROI SPECT variables was performed using the Statistical Package for the Social Sciences (SPSS), version 16. Categorical demographic and clinical data were analysed using chi-square or Fisher exact tests. The normality of continuous demographic, neuropsychological and ROI SPECT and MRI data was assessed based on examination of Q-Q probability plots. Normally distributed data were analysed using independent sample t-tests or univariate analysis of covariance (ANCOVA). Mann Whitney U tests were performed for non-normally distributed data.

#### SPECT analysis

Multivariate ANCOVA (MANCOVA) was used to compare perfusion ratios within the identified candidate ROIs between the VHF-DLB and CBS groups. Covariates were included if they were found to be different between the groups being compared. We subsequently performed an SPM analysis comparing the VHF-DLB group to the CBS group to confirm the findings of our ROI approach.

#### MRI analysis

Independent samples t-tests were performed with cortical ROI volumes and thickness included as dependent variables and with group (that is, CBS versus VHF-DLB) as the independent variable. ROI volume and thickness measurements that showed significant between group differences on the t-tests were then incorporated into a MANCOVA model as dependent variables and with group (that is, CBS versus VHF-DLB) as the independent variable. The following covariates were included: sex, years of education and total intracranial volume. A *post-hoc* Bonferroni correction procedure was applied to adjust for effects of multiple testing.

### Logistic regression analysis

Neuropsychological and imaging measures that were found to be different between the CBS and VHF-DLB groups were then entered as independent variables into a logistic regression model with group membership as the dependent variable.

### Pathological assessment

Three DLB and nine CBS cases came to autopsy. The brains were prepared according to standard neuropathologic practices. Sections from 50 paraffin blocks were examined. Stains and immunostains included Luxol Fast Blue-Hematoxylin and Eosin (LFB-H & E), hyperphosporylated Tau, beta-amyloid, ubiquitin, alpha-synuclein, p62, glial fibrillary acidic protein (GFAP), CD2, Prussian blue and TDP-43.

## Results

### Demographic data

The demographic characteristics of the VHF-DLB, CBS and their respective control groups are shown in Table 1. Years of education was used as a covariate in the SPECT comparison of CBS versus controls because it was higher in the control group. Years of education and sex were used as covariates in all analyses relating to the comparison between the CBS and VHF-DLB groups given that they were different between the two patient groups and given that perfusion differences between men and women have been previously demonstrated using 99mTc-ECD SPECT [54].

**Table 1:** **Demographics of patients with corticobasal syndrome (CBS), non-hallucinating dementia with Lewy bodies (VHF-DLB) and respective matched control groups**

**Demographic and clinical features**	**CBS**	**CBS controls**	**VHF-DLB**	**VHF-DLB controls**
	**(number =31)**	**(number =31)**	**(number =30)**	**(number =30)**
Gender	19 F 12 M^a^	19 F 12 M	10 F 20 M^a^	10 F 20 M
Handedness	29R 2 L	29R 2 L	29R 1 L	28R 2 L
Age of onset (mean ± SEM years)	64.7 ± 1.6	N/A	68.6 ± 1.3	N/A
Age at investigation (mean ± SEM years)	68.5 ± 1.7	70.0 ± 1.2	72.3 ± 1.7	73.1 ± 1.2
Duration of symptoms (mean ± SEM years)	3.8 ± 0.4	N/A	3.7 ± 0.4	N/A
Years of education (mean ± SEM years)^b^	12.4 ± 0.6^bc^	14.5 ± 0.5^b^	14.6 ± 0.7^c^	15.17 ± 0.6
Body side most affected	16R 15 L	N/A	N/A	N/A

^a^Fisher’s Exact Test, *P* =0.01; ^b^t[60] = −2.7, *P* =0.008; ^c^t[60] =2.4, *P* =0.02. F, female; L, left; M, male; N/A, not applicable; R, right; SEM, standard error of the mean.

### Clinical and pathological features

Table 2 displays the clinical features of the DLB cohort at the time of entry into the study. Of the ten cases of possible DLB, eight presented with spontaneous parkinsonism and dementia while two presented with fluctuating attention/alertness and dementia. Of the 20 cases of probable DLB, ten presented with parkinsonism and marked fluctuations in attention and alertness; four exhibited parkinsonism, fluctuating attention/alertness and rapid eye movement (REM) sleep behaviour disorder; two exhibited parkinsonism and REM sleep behaviour disorder; two exhibited parkinsonism and neuroleptic sensitivity; one presented with fluctuating attention/alertness and REM sleep behavior disorder; and one exhibited fluctuating attention/alertness and REM sleep behavior disorder, all in the context of dementia. Of the core diagnostic features of DLB, parkinsonism was the most common at presentation (87%, 26/30 patients). Importantly, all DLB subjects went on to develop parkinsonism within one year of the initial investigation. As a result, two possible DLB cases met criteria for probable disease on longitudinal assessment bringing the total number of probable cases to 22. Of the parkinsonian features, rigidity and gait disturbance were the most prevalent. Fluctuating attention and alertness, also a core diagnostic feature was found in 60% (18/30) of the subjects. Visual hallucinations were not observed in this DLB cohort, which was the basis for their inclusion in this study. Nineteen of the 30 VHF-DLB patients were on cholinesterase inhibitors at the time of this study. However, none had exhibited visual hallucinations prior to initiation of therapy.

**Table 2:** **Clinical characteristics of patients with VHF-DLB at the time of entry into the study**

**Clinical characteristics**	**Frequency (%) at investigation (N =30)**	**Frequency (%) at follow-up (N =30)**
**Core features**		
Parkinsonism	26 (86.7)	30 (100%)^a^
Rigidity	24 (80.0)	-
Gait disturbance	21 (70.0)	-
Bradykinesia	20 (66.7)	-
Tremor	17 (56.7)	-
Postural instability	15 (50.0)	-
Hypomimia	15 (50.0)	-
Fluctuating cognition	18 (60.0)	18 (60.0)
Visual hallucinations	0 (0)	0 (0)
**Supportive features**		
REM sleep behaviour disorder	7 (23.3)	7 (23.3)
Neuroleptic sensitivity	3 (10.0)	3 (10.0)^b^
**Suggestive features**		
Depressive symptoms	14 (46.7)	14 (46.7)
Systematised delusions	7 (23.3)	7 (23.3)
Orthostatic hypotension	5 (16.7)	5 (16.7)
Non-visual hallucinations	5 (16.7)	5 (16.7)
Syncope	1 (3.3)	1 (3.3)

^a^All patients developed parkinsonism within one year of investigation; ^b^neuroleptic use avoided given DLB diagnosis. Only three patients were exposed to an antipsychotic and all had sensitivity. N, number; REM, rapid eye movement; VHF-DLB, visual hallucination-free dementia with Lewy bodies.

The clinical features of the CBS group are shown in Table 3. Asymmetric apraxia (90%; 28/31) and rigidity (90%; 28/31) were the most common features in this cohort initially, followed by language disturbance (77%; 24/31) and early dementia (71%; 2/31). Sixteen patients (51.6%) presented with the right side of their body most affected while 15 (48.4%) had a left-sided presentation. We retrospectively applied the new diagnostic criteria for CBS [55]. Sixteen of our CBS cases met the new criteria for probable CBS while 15 met possible criteria.

**Table 3:** **Clinical characteristics of CBS sample**

**Clinical characteristics**	**Frequency (%) at time of investigation (N =31)**	**Frequency (%) at follow-up (N =31)**
**Extrapyramidal features**		
Rigidity (asymmetric)	28 (90.3%)	31 (100%)
Dystonia	16 (51.6%)	18 (58.1%)
Levodopa trial with poor response^a^	13 (41.9%)	13 (41.9%)
Tremor – postural/action	8 (25.8%)	11 (35.6%)
**Cortical features**		
Apraxia	28 (90.3%)	31 (100%)
Cortical sensory loss	19 (61.3%)	19 (61.3%)
Alien-limb phenomenon	1 (3.2%)	3 (9.7%)
Limb levitation	7 (22.6%)	10 (32.3%)
Myoclonus	9 (29.0%)	13 (41.9%)
Early dementia	22 (71.0%)	22 (71.0%)
Language disturbance	24 (77.4%)	24 (77.4%)

^a^Thirteen patients had a trial of levodopa and all responded poorly based on clinical assessment. Average time for emergence of additional symptoms or signs on follow-up was 1.0 ± 0.3 years. CBS, corticobasal syndrome; N, number.

**Figure 2:**
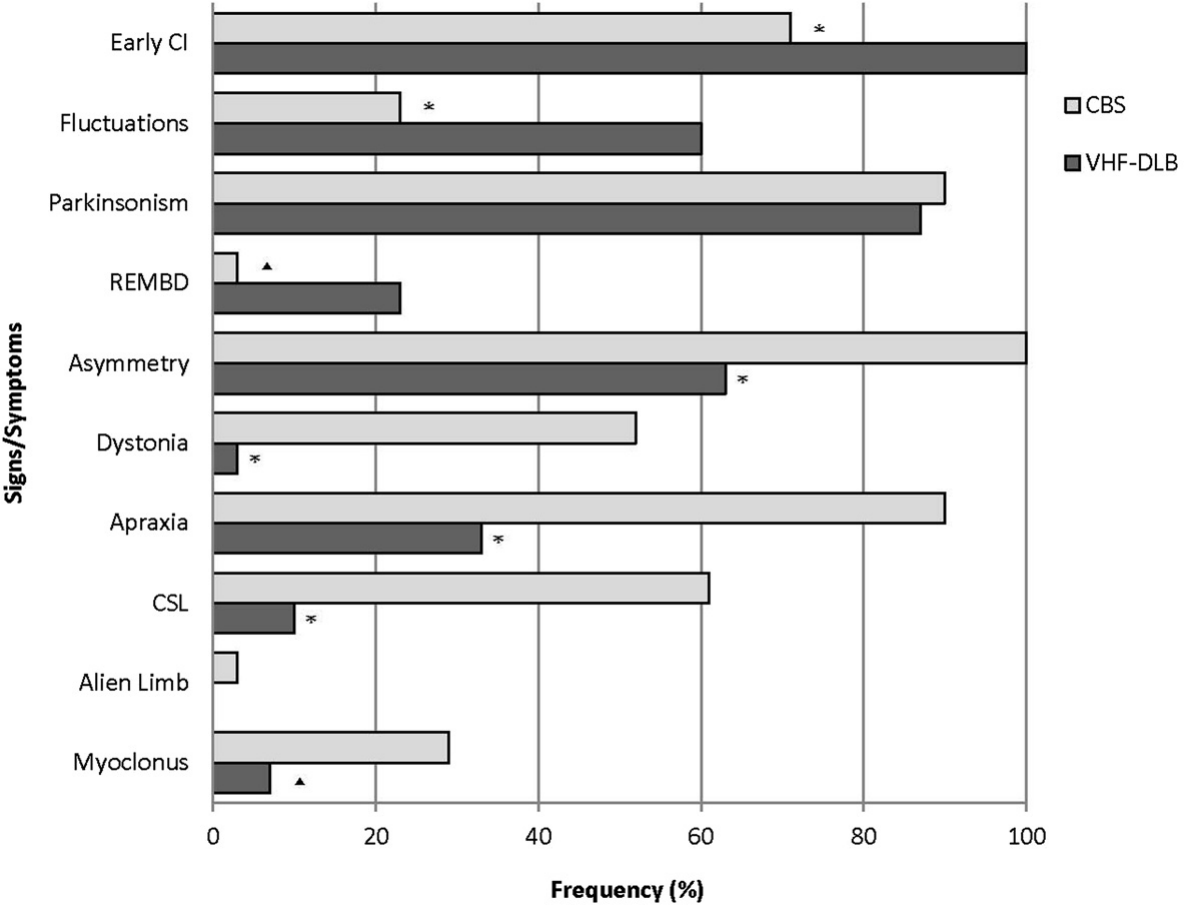
**Bar graph comparing frequency of core signs and symptoms in the CBS versus VHF-DLB groups.** *P <0.005; ▲*P* <0.05. CBS, corticobasal syndrome; VHF-DLB, visual hallucination-free dementia with Lewy bodies.

Figure 2 demonstrates the frequency of the core signs and symptoms of each of the clinical diagnostic categories across both the VHF-DLB and CBS groups. As expected based on the design of this study, there were several differences in the frequency of clinical signs and symptoms between the VHF-DLB and CBS groups. Specifically, asymmetry of the apraxia and/or motor signs, and presence of apraxia, dystonia, myoclonus and cortical sensory loss were more frequent in the CBS group. There were no differences between the presence of parkinsonism or true alien limb phenomenon noted although the latter was an uncommon feature in our CBS group. The VHF-DLB group had a higher occurrence of early cognitive impairment, fluctuations in attention and/or alertness and the presence of REM behavioral disorder.

Subsequent pathology was obtained in three subjects in the VHF-DLB group, all of whom had pathological confirmation of Lewy body disease with all demonstrating concomitant Alzheimer’s pathology and small vessel arteriosclerosis. Nine subjects in the CBS group came to autopsy with pathological diagnoses of pure CBD in five, mixed disease - dementia with agyrophilic grains + CBD + cerebral amyloid angiopathy in one, PSP in two, and FTLD-U/TDP43 proteinopathy due to mutation in the progranulin gene (*GRN*) in one [56]. None of the CBS cases had Lewy bodies, Lewy neurites or other alpha-synuclein positive inclusions.

### Neuropsychological data

The complete neuropsychological, neuropsychiatric and functional profiles of both patient groups are shown in Table 4. The CBS and VHF-DLB groups did not differ on the MMSE or DRS. After controlling for effects of sex and years of education, the VHF-DLB group demonstrated more marked impairment compared to the CBS group on the CVLT-long delay free recall (mean ± SEM =2.4 ± 0.5 versus 6.6 ± 0.6, respectively; F (1,42) =23.1, *P* <0.001) and CVLT-long delay cued recall (mean ± SEM =3.9 ± 0.5 versus 7.4 ± 0.6, respectively; F (1,42) =14.0, *P* =0.001) as well as the WMS-R delayed visual reproduction (mean ± SEM =4.2 ± 1.2 versus 10.5 ± 2.3, respectively; F (1,36) =9.7, *P* =0.004). Because of the observed CVLT differences, we performed secondary analyses to determine if the impairments seen in the VHF-DLB group on recall were due to encoding or retrieval deficits. Both groups demonstrated a similar benefit from cueing on the CVLT (data not shown). However, using the sum of the total number of words retained after each learning trial (one through five), the VHF-DLB group was found to perform more poorly on encoding than the CBS group (VHF-DLB: 21.6 ± 2.4 versus CBS: 31.3 ± 2.0; F (1,42) =8.5, *P* =0.006).

**Table 4:** **Mean (± SEM) scores on neuropsychological, neuropsychiatric and functional measures in the CBS and VHF-DLB groups**

**Psychometric measures**	**CBS (number)**	**VHF-DLB (number)**
**General cognition**		
MMSE/30 (number =59)	21.7 ± 1.2 (31)	22.1 ± 1.1 (28)
Clock Drawing Test/10 (number =26)	6.9 ± 0.8 (9)	7.7 ± 0.6 (17)
NART/127.8 (number =38)	107.5 ± 1.9 (19)	108.4 ± 2.1 (19)
Raven’s Progressive Matrices (number =40)	21.9 ± 1.6 (22)	21.2 ± 1.3 (18)
DRS/144 (number =53)	113.5 ± 4.1 (26)	114.6 ± 3.7 (27)
**Memory**		
CVLT Long Delay Free Recall/16 (number =46)^a^	6.6 ± 0.6 (21)	2.4 ± 0.5 (25)
CVLT Long Delay Cued Recall/16 (number =46)^a^	7.4 ± 0.6 (21)	3.9 ± 0.5 (25)
Delayed Visual Reproduction /41 (number =40^b^	10.5 ± 2.3 (19)	4.2 ± 1.2 (21)
**Language**		
WAB total/100 (number =38)	85.7 ± 2.5 (23)	86.5 ± 2.2 (15)
Boston Naming/30 (number =47)	24.0 ± 1.0 (22)	22.5 ± 1.1 (25)
Semantic Fluency/20 (number =53)	9.8 ± 1.2 (26)	10.3 ± 1.0 (27)
**Praxis**		
WAB praxis/60 (n =48)	53.2 ± 1.6 (27)	55.6 ± 0.6 (21)
**Attention and working memory**		
Digit span - forward/12 (number =50)	7.0 ± 0.6 (23)	6.8 ± 0.5 (27)
Digit span - backward/12 (number =50)	4.6 ± 0.6 (23)	4.3 ± 0.5 (27)
**Visuospatial abilities**		
Rey Osterieth Complex Figure/36 (number =45)	17.0 ± 3.0 (20)	18.5 ± 2.3 (25)
Benton Line Orientation /30 (number =44)	13.2 ± 2.4 (21)	13.4 ± 2.3 (23)
**Executive functions**		
Phonemic (FAS) fluency (number =47)	19.1 ± 2.7 (21)	22.1 ± 3.2 (26)
Trail Making Test A (time in seconds) (number =42)	108.7 ± 14.7 (19)	101.4 ± 11.5 (23)
Trail Making Test B (time in seconds) (number =31)	214.5 ± 39.7 (13)	193.6 ± 20.9 (18)
WCST categories/6 (number =44)	(22)	(22)
Categories 0 to 1: Counts (%)	11 (50%)	17 (77%)
Categories 2 to 4: Counts (%)	11 (50%)	5 (23%)
WCST perseverative errors (number =44)	11.7 ± 2.9 (22)	19.8 ± 3.4 (22)
**Neuropsychiatric features**		
Cornell Depression (number =54)	22.8 ± 2.6 (30)	16.0 ± 2.7 (24)
Neuropsychiatric Inventory/144 (number =55)	12.1 ± 2.5 (29)	10.5 ± 2.4 (26)
**Functional measures**		
DAD (%) (number =57)	68.8 ± 5.7 (30)	78.9 ± 4.1 (27)

^a^ *P* <0.005; ^b^ *P* <0.05. The number of patients tested is listed next to individual measures. Missing data are secondary to the inability of the patient to complete the test. CBS, corticobasal syndrome; CVLT, California Verbal Learning Test; DAD, Disability Assessment Scale for Dementia; DRS, Mattis Dementia Rating Scale; FAS, F-, A-, and S-phonemic fluency; MMSE, Folstein’s Mini-Mental State Exam; NART, National Adult Reading Test; SEM, standard error of the mean; VHF-DLB, visual hallucination-free dementia with Lewy bodies; WAB, Western Aphasia Battery; WCST, Wisconsin Card Sort Test.

### SPM and ROI SPECT analysis

**Figure 3:**
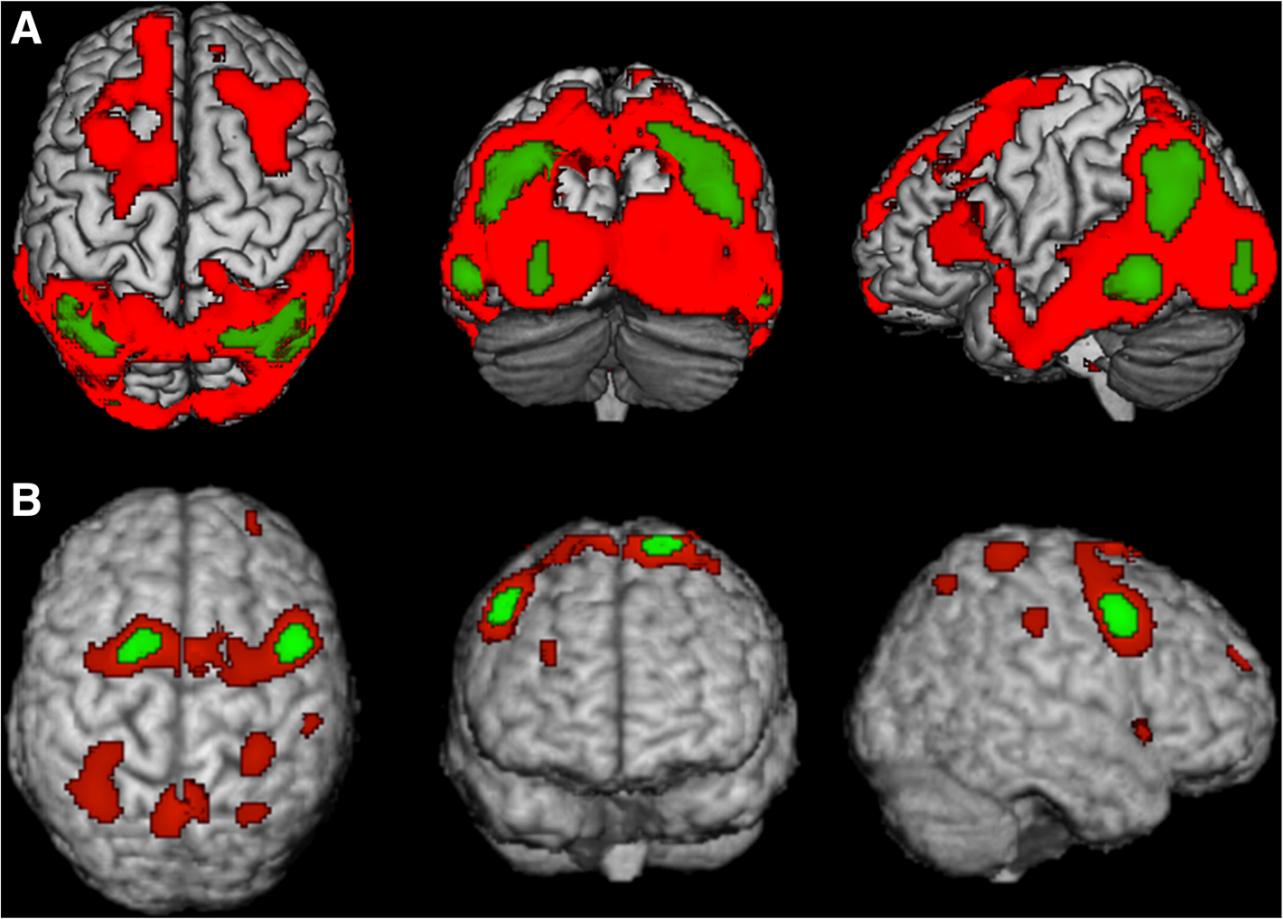
**Statistical Parametric Map (SPM) depicting regions of decreased perfusion in A) VHF-DLB and B) CBS relative to controls.** Red areas are corrected for multiple testing using the False Discovery Rate, while green areas are corrected using the more conservative Family Wise Error method. Views are shown in standard anatomical orientation.

Figure 3 shows the voxel clusters for which a significant reduction in perfusion in the CBS and VHF-DLB groups were found relative to normal controls. The SPM analysis revealed significant reductions in perfusion after correcting the FWE (most conservative) in the right superior frontal gyrus and left middle frontal gyrus in the CBS group relative to controls (Table 5). Analysis after correcting the FDR (less conservative) revealed bilateral hypoperfusion in the medial and dorsolateral frontal area, and parietal regions in the CBS groups relative to controls (Table 5). The VHF-DLB group demonstrated significant reductions in the left and right middle temporal gyri, right superior parietal lobule, left precuneus, left superior temporal gyrus, left inferior parietal lobule and left middle occipital gyrus relative to controls (FWE-corrected) (See Table 6). Less conservative correction methods using the FDR revealed the same regions of reduced perfusion as demonstrated by FWE-correction methods in addition to symmetrical, bilateral hypoperfusion in frontal and temporal regions and in the left caudate in VHF-DLB compared to controls (Table 6). There were no areas of increased perfusion seen in the controls relative to both patient groups.

**Table 5:** **Areas of relative hypoperfusion on SPECT when CBS compared to their respective control groups**

**Anatomical locus (Brodmann area)**	**Talairach coordinates**			**Number of voxels**	**SPM t-score (** ***P*** **-value)**
	**x**	**y**	**z**		
**CBS versus controls (FWE-corr)**					
Right middle frontal gyrus (6)	50	8	42	236	5.4 (*P* =0.003)
Left superior frontal gyrus (6)	−18	9	70	99	5.4 (*P* =0.005)
**CBS versus controls (FDR-corr)**					
Right middle frontal gyrus (6)	50	8	42	1791	5.4 (*P* =0.005)
Right superior frontal gyrus (6)	6	7	70		4.7 (*P* =0.007)
Right sub-gyrus (6)	30	−1	55		4.0 (*P* =0.014)
Left superior frontal gyrus (6)	−18	9	70	850	5.4 (*P* =0.005)
Left superior frontal gyrus (6)	0	−2	68		3.5 (*P* =0.020)
Left middle frontal gyrus (6)	−26	−1	50		3.6 (*P* =0.019)
Left precentral gyrus (9)	−34	9	31	203	4.4 (*P* =0.009)
Left precuneus (7)	−2	−57	62	655	4.4 (*P* =0.009)
Right precuneus (7)	4	−53	65		4.4 (*P* =0.010)
Left postcentral gyrus (7)	−4	−51	67		3.7 (*P* =0.017)
Left superior parietal lobule (7)	−34	−53	60	834	4.1 (*P* =0.013)
Left superior parietal lobule (7)	−32	−46	47		3.8 (*P* =0.017)
Left postcentral gyrus (2)	−30	−37	70		4.0 (*P* =0.015)
Right postcentral gyrus (2)	34	−35	68	213	4.1 (*P* =0.013)
Right postcentral gyrus (2)	48	−27	40	163	3.9 (*P* =0.015)
Right inferior frontal gyrus (47)	44	17	−3	96	3.8 (*P* =0.016)
Right superior frontal gyrus (10)	32	57	23	31	3.8 (*P* =0.017)
Right superior parietal lobule (7)	32	−59	58	67	3.7 (*P* =0.017)

CBS, corticobasal syndrome; FDR-corr, corrected for False Discovery Rate; FWE-corr, corrected for Family-Wise Error; SPECT, single-photon emission computed tomography; SPM, Statistical Parametric Mapping.

**Table 6:** **Areas of relative hypoperfusion on SPECT when VHF-DLB is compared to their respective control group**

**Anatomical locus (Brodmann area)**	**Talairach coordinates**			**Number of voxels**	**SPM t-score (** ***P*** **-value)**
	**x**	**y**	**z**		
**VHF-DLB versus controls (FWE-corr)**					
Left middle temporal gyrus (21)	−57	−49	−3	413	6.2 (*P* =0.000)
Right middle temporal gyrus (39)	44	−63	31	1292	5.9 (*P* =0.001)
Right superior parietal lobule (7)	30	−64	46		5.6 (*P* =0.003)
Left precuneus (19)	−34	−64	42	1195	5.9 (*P* =0.001)
Left superior temporal gyrus (39)	−48	−57	27		5.7 (*P* =0.002)
Left inferior parietal lobule (40)	−46	−56	39		5.5 (*P* =0.004)
Right middle temporal gyrus (21)	61	−35	−10	211	5.4 (*P* =0.005)
Left middle occipital gyrus	−28	−89	−2	100	5.1 (*P* =0.013)
**VHF-DLB versus controls (FDR-corr)**					
Left middle frontal gyrus (21)	−57	−49	−3	33550	6.2 (*P* =0.000)
Left precuneus (19)	−34	−64	42		5.9 (*P* =0.000)
Right middle temporal gyrus (39)	44	−63	31		5.9 (*P* = 0.000)
Right middle frontal gyrus (8)	36	16	45	1743	3.9 (*P* =0.002)
Right middle frontal gyrus (9)	36	27	37		3.9 (*P* =0.002)
Right superior frontal gyrus (8)	20	39	39		3.7 (*P* =0.003)
Left medial frontal gyrus (11)	−4	61	−15	144	3.7 (*P* =0.003)
Right inferior frontal gyrus (47)	50	27	−3	344	3.6 (*P* =0.004)
Left sub-lobar caudate	−14	10	5	41	3.4 (P =0.006)
Right superior frontal gyrus (9)	14	52	25	23	3.2 (*P* =0.008)

FDR-corr ,corrected for False Discovery Rate; FWE-corr, corrected for Family-Wise Error; SPECT, single-photon emission computed tomography; SPM, Statistical Parametric Mapping; VHF-DLB, visual hallucination-free dementia with Lewy bodies.

We then examined for perfusion differences between the VHF-DLB and CBS groups directly using the pre-specified hypothesis that areas differentiating the two patient groups will reside within non-overlapping regions of reduced perfusion identified in their respective case versus control comparisons (Figure 1). Using a MANCOVA model controlling for effects of sex and years of education with the independent variable being patient group and the dependent variables being perfusion ratios in ROIs, the left occipital pole was found to be the only ROI showing reduced perfusion in the VHF-DLB patients compared to the CBS group (*F* (19,39) =2.66, *P* =0.005; Wilk’s Λ =0.44, partial η^2^ = 0.56). The corrected mean perfusion ratio ± SEM for the VHF-DLB group was 0.80 ± 0.02 versus 0.88 ± 0.02 for the CBS group (F (1,57) =7.4, *P* =0.009, R^2^ = 0.13 (*post-hoc* univariate analysis)). No other ROIs showed differential perfusion between the patient groups. This finding was subsequently confirmed using SPM. Specifically, after controlling for the effects of years of education and sex in SPM, the left cuneus (Brodmann Area 18; Talairach coordinates x = −14, y = −96, and z =4; number of voxels in the cluster =159) was found to show reduced perfusion in the VHF-DLB group compared to the CBS group (SPM t-score =3.9, *P* <0.001 [uncorrected]).

### MRI ROI cortical volumetric and thickness analysis

Several bilateral cortical ROI volume measures and one cortical ROI thickness measure within frontal, temporal and parietal regions were found to be reduced in the CBS group compared to the VHF-DLB group in the independent samples t-test analysis. These ROI volumetric and thickness measures were incorporated into a MANCOVA analysis and the overall model that controlled for sex, years of education, and total intracranial volume did not show any statistically significant differences between the VHF-DLB and CBS groups with respect to cortical ROI volume and thickness measures.

### Logistic regression predicting group membership

A logistic regression was performed to determine the effects of CVLT-long delay free recall score and perfusion within the left occipital pole on predicting patient group membership (that is, VHF-DLB versus CBS). So as to not violate the assumption of independence of observations required for logistic regression, the CVLT-long delay cued recall and WMS-R delayed visual reproduction were not included in this model because they were strongly correlated with the CVLT-long delay free recall (data not shown). The overall logistic regression model was statistically significant (χ^2^ [2] =23.7, *P* <0.0005). The model correctly classified 80.4% of cases and accounted for 53.9% (Nagelkerke *R*^*2*^) of the variance in patient group membership. CVLT-long delay free recall was the only independent variable that correctly predicted group membership (Wald test [1] =10.7, *P* =0.001). Specifically, worse performance on this memory test was associated with an increased likelihood of belonging to the VHF-DLB group.

## Discussion

This study demonstrated that neuropsychological measures assessing both verbal and visual episodic memory combined with perfusion in the left occipital region may be useful in distinguishing between VHF-DLB and CBS. Specifically, we have found that the VHF-DLB group performed worse on the CVLT long delay free and cued recall as well as on the WMS-R delayed visual reproduction task and showed decreased perfusion in the left occipital pole ROI compared to CBS patients. However, in a logistic regression analysis, the CVLT long delay free recall scores had better predictive value than left occipital pole perfusion in terms of determining group membership. The CBS and VHF-DLB groups did not differ on MMSE or DRS, suggesting that they were well-matched for dementia severity, which was in the mild stages in both groups. Both patient groups also had a similar duration of disease at the time of investigation, therefore, making it unlikely to be a confounding factor.

CBS and DLB groups often exhibit primarily executive dysfunction greater than memory impairment, exemplified by disproportionate deficits when performing the WCST, Stroop test, TMT, and Delis-Kaplan Executive Function System [57–60]. Visuospatial dysfunction has also been shown to be disproportionately severe in DLB patients, when assessed using standardised measures such as the Benton Judgment of Line Orientation (BJLO) or the Rey-Ostereith Complex Figure Copy Paradigm (ROCF-CP) [61]. Such deficits have been attributed to problems with perceptual processing and apraxia in DLB patients [62,63]. When compared to PSP and Multiple System Atrophy (MSA), CBS has been shown to have the most consistent and severe impairment of visuospatial function on the BJLO and the Visual Object and Space Perception Battery (VOSP) [64,65]. Indeed, tests assessing general cognition, attention and working memory, executive functions, language, praxis and visuospatial abilities did not distinguish between the VHF-DLB and CBS groups included in this study indicating substantial overlap in their overall clinical neuropsychological profiles.

The CVLT is a standardized and validated test of verbal episodic memory function and is a widely used measure in the dementia literature [66]. Our results suggest that although CBS and VHF-DLB patients both benefit similarly from cueing, indicating that frontal-subcortical dysfunction contributes to their memory deficits, the VHF-DLB group recalled fewer words overall compared to the CBS group. This may be due to more severe encoding versus retrieval problems in the VHF-DLB group. The sum of the total number of words retained after learning trials, one through five, was significantly lower in the VHF-DLB group suggesting that this group had more troubles with encoding than the CBS group. This may be due to concomitant AD pathology in the VHF-DLB group, which is consistent with prior studies showing significant pathological overlap between DLB and AD [1] as well as the finding that three of our VHF-DLB cases that came to autopsy all demonstrated concomitant DLB, AD as well as arteriosclerosis. While the inclusion of possible VHF-DLB cases increases the likelihood that these patients may have AD pathology and not DLB, the fact that all of our VHF-DLB cases had parkinsonism makes pure AD pathology very unlikely in this patient group.

The SPECT data showed typical perfusion profiles of CBS [67–70] and DLB [46], as demonstrated in prior studies. This strongly supports that our patient groups were similar to other published case series and improves on diagnostic accuracy. Areas of relative hypoperfusion have been found to involve frontal and parietal regions in both DLB and CBS [46,69,71]. While bilateral perfusion deficits are characteristic of DLB [72], CBS patients tend to show asymmetrical perfusion syndromes in most [71,73], but not all, studies [69]. These prior studies demonstrate overlap in the hypoperfused brain regions found on SPECT in both DLB and CBS.

Reduced perfusion in the left occipital pole ROI was observed in the VHF-DLB group compared to the CBS group, while there were no brain ROIs with significantly reduced perfusion observed in the CBS relative to the VHF-DLB group. Occipital hypoperfusion most pronounced at the parieto-occipital junction on SPECT is a well-known feature of DLB [46,72] and is one of the supportive features in the consensus diagnostic criteria [9]. Occipital lobe dysfunction in DLB was first demonstrated using fluoro-deoxyglucose positron emission tomography (FDG-PET) [74,75]. It was later shown that occipital lobe abnormalities could be used to differentiate DLB from AD, with a sensitivity and specificity ranging as high as 92% for both [76–78]. Subsequently, occipital lobe dysfunction was confirmed using SPECT tracers including I-labelled isopropyl-iodoamphetamine SPECT [79] and 99 m Tc-HMPAO SPECT [80]. Pasquier *et al*. [81] demonstrated decreased bilateral occipital lobe perfusion in DLB as compared to AD using 99 m Tc-ECD SPECT. Bilateral occipital lobe hypoperfusion in combination with maintained perfusion within the left internal temporal region has been shown to favour a diagnosis of DLB over AD with 65% sensitivity and 71% specificity [81,82].

The left occipital pole ROI in our SPECT template contained 712 voxels, mainly in BA 18 and part of BA 17, comprising secondary visual association and the primary visual cortices, respectively. This ROI includes the following neuroanatomical structures that function together to interpret the visual world: cuneus, lingual gyrus and lateral occipital gyrus. A more conservative SPM analysis confirmed that the left cuneus showed significant reductions in perfusion in the VHF-DLB group compared to the CBS patients. The cuneus is involved in primary visual processing, particularly in integrating ocular position signals to process stimuli position in space [83]. It has also been shown to have a variety of roles within the cognitive domain, including response inhibition [84], working memory [85] and behavioural engagement in cognitive control [86]. The lingual gyrus is involved in visual memory [87], encoding of complex images [88] and identification/recognition of letters and words [89,90]. A more recent resting state functional MRI study also identified a role of the bilateral lingual gyri in object colour knowledge [91,92]. The lateral occipital complex may play an important role in the recognition and perception of objects [93]. Many of these processes are selectively impaired in individuals suffering from DLB.

Our study had both strengths and limitations that warrant discussion. A main strength was our relatively large sample size given the rarity of both diagnostic groups included. An important limitation is the lack of pathological confirmation in the majority of our cases. However, a portion of each group was confirmed with the predicted pathology. All three of the VHF-DLB cases were confirmed to have DLB, and six of the nine CBS subjects autopsied had pathologic verification of CBD, a ratio consistent with previous studies [5]. The remaining CBS cases had pathology recognized to cause a CBS and none had additional Lewy body or other alpha-synuclein-related pathology. To overcome the lack of pathological confirmation in our sample, we chose as a first step in this line of investigation to include individuals meeting consensus diagnostic criteria for CBS and VHF-DLB. We acknowledge that this may reduce the chances that our findings are generalisable to earlier clinical stages of VHF-DLB and CBS. Therefore, we will plan to validate our findings in an independent cohort of pathologically confirmed cases that is being ascertained with both diagnoses and that have been followed from early stages using CVLT and brain SPECT when they are particularly hard to distinguish from each other. Thus, these results represent the first phase of an ongoing project. Importantly though, we demonstrate that many of the VHF-DLB patients have overlapping clinical features with the CBS group and vice versa. An additional limitation is the reduced statistical power for the neuroimaging analyses, which is likely the reason why the perfusion differences between the VHF-DLB and CBS groups localised only to the left occipital region and not to the corresponding contralateral side and why the MRI volumetric and thickness measures did not show any differences between the patient groups. This is especially the case for the analysis of MRI data as there were only 21 useable MRIs in the CBS group and 23 in the VHF-DLB group. Despite this limitation, our results suggest that perfusion measures may be more sensitive than atrophy measures in syndromes that have some degree of clinical overlap. This is supported by prior research of focal cortical atrophy syndromes, such as FTLD, whereby perfusion reductions on SPECT are more extensive than atrophy detected on MRI in the early stages of disease and in longitudinal follow-up, indicating increased sensitivity of this modality as a potential biomarker [94–96].

## Conclusions

In spite of the limited pathology available, the benefit of our study is that it provides neuropsychological and neuroimaging features useful for differentiating VHF-DLB and CBS in the antemortem period. Indeed, the diagnostic challenges faced by clinicians are in the antemortem period, when only syndromic presentations, and a lack of any pathological verification, are available to inform their diagnostic considerations. Our segregation of VHF-DLB and CBS groups, based on expert consensus and strongly supported by SPECT perfusion profiles, represents the same information that clinicians will need to rely on to inform treatment decisions, without the guidance of pathology. Therefore, our study provides clinicians with information they can use in difficult cases to help support diagnostic stratification and treatment plans for these two overlapping clinical entities.

## Abbreviations

ANCOVA: analysis of covariance
BA: Brodmann area
BJLO: Benton Judgment of Line Orientation
BNT: Boston Naming Test
CBD: corticobasal degeneration
CBS: corticobasal syndrome
CSDD: Cornell Scale for Depression in Dementia
CSF: cerebrospinal fluid
CVLT: California verbal learning test
DAD: Disability Assessment for Dementia
DLB: dementia with Lewy bodies
DRS: Dementia Rating Scale
FDG-PET: fluoro-deoxyglucose positron emission tomography
FDR: false discovery rate
FMRIB: functional magnetic resonance imaging of the brain
FOV: field of view
FTLD: frontotemporal lobar degeneration
FWE: family-wise error
FWHM: full width at half maximum
GFAP: glial fibrillary acidic protein
LFB-H & E: luxol fast blue-haematoxylin and eosin
MANCOVA: multivariate analysis of variance
MMSE: Mini-Mental State Exam
MNI: Montreal Neurologic Institute
MRI: magnetic resonance imaging
MSA: multiple system atrophy
NART-R: National Adult Reading Test-Revised
NEX: number of excitations
NPI: Neuropsychologic Inventory
PD: proton density
PSP: progressive supranuclear palsy
rCBF: regional cerebral blood flow
REM: rapid eye movement
ROCF-CP: Rey-Ostereith Complex Figure Copy Paradigm
ROI: region of interest
SPECT: single-photon emission computed tomography
SPGR: spoiled gradient
SPM: statistical parametric mapping
SPSS: Statistical Package for the Social Sciences
TDP-43: TAR DNA binding protein 43
TE: echo time
TMT-A and –B: Trail Making Test A and B
TR: repetition time
VHF-DLB: visual hallucination-free dementia with Lewy bodies
VOSP: Visual Object and Space Perception Battery
WAB: Western Aphasia Battery
WCST: Wisconsin Card Sort Test
WMS-R: Wechsler Memory Scale-Revised
